# Validation of the Communication Profile-Adapted in Ethiopian children with neurodevelopmental disorders

**DOI:** 10.1017/gmh.2021.44

**Published:** 2021-12-13

**Authors:** Caterina Ceccarelli, Ioannis Bakolis, Bethlehem Tekola, Mersha Kinfe, Anton Borissov, Fikirte Girma, Rehana Abdurahman, Tigist Zerihun, Charlotte Hanlon, Rosa A. Hoekstra

**Affiliations:** 1 Department of Psychology, Institute of Psychiatry, Psychology and Neuroscience, King's College London, London, UK; 2 Department of Biostatistics, Institute of Psychiatry, Psychology and Neuroscience, King's College London, London, UK; 3 Centre for Implementation Science, Health Services and Population Research Department, Institute of Psychiatry, Psychology and Neuroscience, King's College London, London, UK; 4 Department of Psychiatry, WHO Collaborating Centre for Mental Health Research and Capacity-Building, School of Medicine, College of Health Sciences, Addis Ababa University, Addis Ababa, Ethiopia; 5 Department of Psychiatry, Yekatit 12 Hospital and Medical College, Addis Ababa, Ethiopia; 6 Department of Psychiatry, St. Paul's Hospital Millennium Medical College, Addis Ababa, Ethiopia; 7 Centre for Innovative Drug Development and Therapeutic Trials for Africa (CDT-Africa), College of Health Sciences, Addis Ababa University, Addis Ababa, Ethiopia; 8 Centre for Global Mental Health, Department of Health Services and Population Research and WHO Collaborating Centre for Mental Health Research and Capacity-Building, Institute of Psychiatry, Psychology and Neuroscience, King's College London, London, UK; 9 King's Global Health Institute, King's College London, London, UK

**Keywords:** Caregiver, communication disorders, neurodevelopmental disorders, psychometrics, sub-Saharan Africa

## Abstract

**Background:**

Neurodevelopmental disorders (NDDs) are conditions affecting a child's cognitive, behavioural, and emotional development. Appropriate and validated outcome measures for use in children with NDDs in sub-Saharan Africa are scarce. The aim of this study was to validate the Communication Profile Adapted (CP-A), a measure developed in East Africa to assess caregivers' perception of communication among children with NDDs.

**Methods:**

We adapted the CP-A for use in Ethiopia, focusing on the communicative mode (CP-A-mode) and function (CP-A-function) scales. The CP-A was administered to a representative sample of caregivers of children with NDDs and clinical controls. We performed an exploratory factor analysis and determined the internal consistency, test-retest reliability, within-scale, known-group, and convergent validity of the identified factors.

**Results:**

Our analysis included *N* = 300 participants (*N* = 139 cases, *N* = 139 clinical controls, *N* = 22 who did not meet criteria for either cases or controls). Within the CP-A-mode, we identified two factors (i.e. *verbal* and *physical communication*); the CP-A-function scale was unidimensional. Combining both scales into one summary variable (the CP-A-total) resulted in a scale with excellent internal consistency and test-retest reliability (Cronbach's alpha = 0.97; Kappa = 0.60–0.95, *p* < 0.001; ICC = 0.97, *p* < 0.001). Testing known-group validity, the CP-A-total scores were significantly higher for controls than cases (Δ mean = 33.93, *p* < 0.001). Convergent validity assessment indicated that scores were negatively and moderately correlated with clinical severity (*ρ* = −0.25, *p* = 0.04).

**Conclusion:**

The CP-A is a valid tool for the assessment of communication among children with NDDs in Ethiopia. It holds promise as a brief, quantitative, and culturally appropriate outcome measure for use in sub-Saharan Africa.

## Background

Neurodevelopmental disorders (NDDs) are a group of conditions that affect a child's cognitive, behavioural, and emotional development which include intellectual disability (ID), autism spectrum disorder (ASD), and attention deficit hyperactivity disorder (ADHD) (World Health Organization, 2019). According to Global Burden of Disease (GBD) 2016 estimates, developmental disabilities, including NDDs, affect 52.9 million children under 5 years of age. Of these, 95% live in low and middle-income countries (LMICs). Nearly 15 million of these children live in sub-Saharan Africa (Olusanya *et al*., 2018). Ethiopia is one of the top 10 nations globally, with an estimated 1.3 million children living with developmental disabilities (Olusanya *et al*., 2018). Children affected by NDDs and their caregivers experience severe stigma and social exclusion; this is especially true for those with ID and/or ASD (Tekola *et al*., 2016, 2020; Tilahun *et al*., 2016).

Despite the high prevalence and burden of NDDs, there is a wide gap between needs and service provision in sub-Saharan Africa. This applies to both fund allocation and the availability of trained personnel (World Health Organization, 2013; Strand *et al*., 2016; Chisholm *et al*., 2019). Ethiopia, with a population of nearly 110 million, is served by only one specialist child psychiatrist. There are 0.08 general psychiatrists and 0.04 psychologists per 100000 people, but these cadres of workers have no specialist expertise in NDDs (World Health Organization, 2018).

This gap in resources and services for children with NDDs extends to research (Patel *et al*., 2013; Tomlinson *et al*., 2014). Only a negligible fraction of research on child development and mental health is conducted in LMICs (Kieling *et al*., 2011; Durkin *et al*., 2015; Nielsen *et al*., 2017; Hoekstra *et al*., 2018). This research gap results in an incomplete and biased body of knowledge (Durkin *et al*., 2015; Hoekstra *et al*., 2018). When evidence-based tools are lacking, diagnosis and intervention initiation tend to occur later or not occur at all, potentially impairing the prognosis and increasing the risk of comorbidities in affected children (Ruparelia *et al*., 2016; Guralnick, 2017).

The development and evaluation of contextually appropriate interventions and harmonised and contextually valid outcome measures are essential to effectively address the service gap (Kieling *et al*., 2011; Tomlinson *et al*., 2014). There is currently no consensus on which outcome measures should be used in the evaluation of interventions targeting NDDs (Kohli-Lynch, Tann and Ellis, 2019). There is an urgent need for tools that are both accessible and appropriate for use in low-resource settings. Such tools are recommended to be: (i) of high quality, (ii) open-source and open-access, (iii) culturally appropriate, (iv) intuitive, (v) brief, (vi) acceptable, and (vii) easy to administer (Prince, 2013; Durkin *et al*., 2015; Ruparelia *et al*., 2016; de Vries, 2016; Carruthers *et al*., 2018; Bakolis *et al*., 2019; Kohli-Lynch *et al*., 2019). These criteria rule out many of the existing outcome measures, which are often prohibitively expensive or rely on administration by highly qualified specialists (Durkin *et al*., 2015).

In this study, we validate two scales of the Communication Profile-Adapted (CP-A) as a brief, culturally appropriate, caregiver-reported outcome measure for Ethiopia. The CP-A assesses caregivers' perceptions of their child's abilities and activities for communication, and participation in family and community events (Bunning *et al*., 2014). It was developed to assess communication in children with complex communication needs and is thus suitable for use in children with NDDs. It has yet to be validated and assessed in its psychometric properties. The CP-A meets important criteria for use in low-resource settings. It has a solid theoretical background, based on the International Classification of Functioning, Disability and Health Framework (World Health Organization, 2001; Hartley and Wirz, 2002; Bunning *et al*., 2014). It also meets the requirement of cultural relevance for sub-Saharan Africa as it was developed in Uganda and Kenya (Baker and Hartley, 1998, 1999; Bunning *et al*., 2014). Because it is an open-access tool, translations and adaptations can be readily made. It is also easy to administer and does not use technical terminology (Bunning *et al*., 2014).

We selected those scales of the CP-A that most closely reflect the key aspects to be targeted in interventional investigations for children with NDDs and hold promise as quantitative scales. These scales focus on the child's communicative mode and function. Both were adapted to the local context and assessed for validity and reliability. We hypothesised that children with NDDs would score significantly lower on the CP-A than controls and that CP-A scores would be negatively correlated with the severity of clinically diagnosed NDDs.

## Methods

### Setting

This study was carried out in Addis Ababa, at Yekatit 12 and St Pauls Millennium Medical College government hospitals between August 2018 and May 2019. The ethics protocol was approved by the College of Health Sciences Institutional Review Board at Addis Ababa University (062/16/Psy) and King's College London (HR-16/17–3489). In addition to validation of the CP-A, the data collection comprised further questionnaires, the validation of which are reported in Borissov *et al*. (2021).

### Participants

Participants were 300 caregivers with long-term responsibilities for children aged 2–9 years, attending either the general paediatric or child mental health clinic of the two hospitals. The paediatric clinics consecutively recruited children with physical health conditions for the clinical control group. The mental health clinics consecutively recruited children with either NDDs alone or with a comorbid mental health condition for the case group, in accordance with the Diagnostic and Statistical Manual of Mental Disorders, Fifth Edition (DSM-5) (American Psychiatric Association, 2013). Given that no standardised diagnostic tests are available in Ethiopia, health practitioners relied on their clinical judgment based on interviews with caregivers, and observations and interactions with children. The clinical diagnoses in the mental health clinic were provided by general psychiatrists without specialist expertise in child psychiatry, as this specialty training is not available in Ethiopia.

Eligible families were approached by the attending clinician at either clinic and given a flyer with information about the study. Data collection and consent taking was done by clinic nurses who worked independently from the clinicians supporting the families. A subgroup of 40 caregivers was invited for a retest of an average of 19.6 days (s.d. = 3.8) after initial test data collection. All participants provided written informed consent.

### Measures

#### CP-A

The CP-A is composed of 51 questions, divided into 3 main sections named ‘body function and structure’, ‘activities for communication’, and ‘participation’. The portion ‘activities for communication’ includes six scales. Two of those, namely communicative mode (CP-A-mode) and communicative function (CP-A-function) were selected for validation in this study, see Tables 2 and 3 for item content. We refer to the combined items of these two scales as the CP-A-total.

Items' responses refer to a 0–4 Likert scale where [0] stands for ‘never’, [1] ‘rarely’, [2] ‘sometimes’, [3] ‘usually’, and [4] ‘always’. Participants were shown a visual ladder representation of this scale with 0 being the lowest and 4 the highest rung (online Supplementary material Fig. S1).

#### Demographic information

Clinician-assigned severity levels were available for a subgroup of participants with a diagnosis of ID (*N* = 24) and ASD (*N* = 50). General psychiatrists working in the two hospitals rated the child's condition, based on the DSM-5 severity ratings, as [1] ‘mild’, [2] ‘moderate’, or [3] ‘severe’. A structured questionnaire was used to collect caregiver-reported demographic information.

### Procedure

#### CP-A adaptation, translation, and pre-testing

The CP-A was translated to Amharic, one of Ethiopia's official languages, and adapted to the local context. The instrument was considered by a consensus committee comprising native Amharic speakers fluent in English with expertise in the field. Following forward and backward translation, the committee met to formulate a version for pre-testing. This draft was pre-tested with 20 participants through cognitive interviews to understand how respondents interpreted instructions and items (Willis, 1999). Feedback was recorded by data collectors and subsequently discussed in the committee to establish a final draft.

Within the CP-A-mode, the content of question 1j was changed to *‘Amharic’* to reflect the main language spoken in Addis Ababa. Item *l*, *Behaviour*, was followed by examples of challenging behaviours in brackets (e.g. crying, shouting) as cognitive interviews indicated that the direct translation of the term ‘behaviour’ was unclear to respondents. The original CP-A response scale comprised 8 rungs of ladders; this was shortened to 4 rungs (online Supplementary material Fig. S1) after cognitive interviewing indicated that participants could not meaningfully differentiate between the small increments of the original ladder. Within the CP-A-function, the original version of the scale did not just require a response using the ladder but also asked the extent to which the item is a problem for the respondent. This second part was removed to improve consistency as well as administration ease and time. In its original format, the CP-A asked both questions on how the child communicates with the respondent, and how the respondent communicates with the child. Caregivers participating in the cognitive interviews had difficulty distinguishing between these questions. In response, the section on how the caregiver communicates with the child was removed, focusing solely on the mode of communication of the child.

#### Data collection and entry

Data collection took place in Yekatit 12 and St Paul's Millennium Medical College general paediatric and child mental health clinics. To allow for the participation of non-literate caregivers, instruments were administered through face-to-face interviews by nurses who had received training in data collection procedures and questionnaire administration. Participating caregivers were reimbursed for their travel costs. Data were double entered using Epidata, version 3.1 (Christiansen and Lauritsen, 2003), to reduce the risk of data errors.

### Data analyses

#### Demographics and item checks

The data were analysed using STATA, version 16 (Stata Corp, 2019). Group differences in demographic variables between cases and controls were explored using unpaired *t* test, Mann–Whitney test, Chi-Square, or Fisher's exact test depending on variables' characteristics. Missing values (*N* = 3) in the CP-A were replaced by median imputation (Zhang, 2016).

#### Exploratory Factor Analysis (EFA)

The factor structure of the (i) CP-A-mode, (ii) CP-A-function, and (iii) CP-A-total were examined with the use of item exploratory factor analysis (EFA) (Bartholomew *et al*., 2008). For each, we evaluated the polychoric matrix of their respective items. Exploratory factor analysis was applied to the matrix of item correlation coefficients, to identify possible underlying dimensions. We used two criteria to aid the choice of the number of factors and provide empirical support for the selection: the scree plot and the criterion of eigenvalues above 1. *χ*^2^ was used as a goodness-of-fit test to evaluate the adequacy of the number of extracted factors (Pett *et al*., 2003). To assist data interpretation promax oblique rotation was used (Bartholomew *et al*., 2008; Baldwin, 2019). Factors were labelled referring to theoretical notions and interpretability.

#### Validity

Our approach of studying the validity and reliability was guided by the consensus-based standards for the selection of health measurement instruments (COSMIN) guidelines (Mokkink *et al*., 2010, 2019).

##### Within-scale validity

We assessed within-scale validity (the extent to which the subscales of an instrument measure the same concept; Brohan *et al*., 2013)) by examining the correlation between summative scores of the identified factors as well as those of the CP-A-mode and CP-A-function.

##### Known-group validity

Known-group validity (i.e. the ability to distinguish among distinct groups; Streiner *et al*., 2015) was tested by assessing group differences between the NDDs and clinical control group across identified factors. Unadjusted and adjusted mean differences between cases and controls were tested using multivariable linear regression, with group membership and previously identified demographic characteristics as independent variables.

##### Convergent validity

Convergent validity (how related different measures assessing associated constructs are; Streiner *et al*., 2015), was assessed by estimating the correlation between the total scores of the identified factors and clinician-rated clinical severity measures available for a subset of children with ID or ASD. The clinical severity scores for each disorder were merged into a single score to maximise sample size; in the case of double diagnoses, the highest severity score was retained.

#### Reliability

Internal consistency (the extent to which items of the same scale measure the same construct) was evaluated through Cronbach's alpha (*α*) (Revicki, 2014). Test-retest reliability (the agreement between scores on the same scale across timepoints) was assessed through weighted Kappa and Interclass Correlation Coefficient (ICC) (Streiner, Norman and Cairney, 2015). The ICC was calculated for continuous summary scores, weighted Kappa for single categorical items.

## Results

### Demographics and item checks

Our sample included *N* = 300 caregivers, comprising *N* = 139 cases and *N* = 139 clinical controls. Children in the case group presented with ASD, ID, ADHD, language delay, global developmental delay, and/or Down's syndrome, see Table 1 (severity ratings details are provided in online Supplementary material Table S1). Those in the clinical control group were affected by a range of physical health problems (e.g. cardiovascular, respiratory, neurological conditions; details in online Supplementary Table S2). The remaining participants (rest: *N* = 22) included children who did not meet the criteria to be in either the case or clinical control group (e.g. they were diagnosed with epilepsy or a mental health condition, but did not have an NDD) and those for whom diagnostic information was incomplete (full details in online Supplementary Table S3).

**Table 1.** Conditions reported in the case group including comorbidities.

Neurodevelopmental disorders (*N* = 139)		
	*N*	%
Condition		
ASD	55	40
ADHD	28	20
ID	19	17
LD	6	4
DS	1	0.7
GDD	1	0.7
ASD + ID	11	8
ASD + ADHD	6	4
ASD + LD	2	1
ASD + GDD	1	0.7
ADHD + ID	2	1
ADHD + LD	1	0.7
ID + DS	2	1
ID + GDD	1	0.7
DS + GDD	1	0.7
ASD + ADHD + ID	1	0.7
ADHD + ID + DS	1	0.7
Total	139	100

*Note*. ASD, autism spectrum disorder; ADHD, attention deficit hyperactivity disorder; ID, intellectual disability; LD, language disorder; DS, Down's syndrome; GDD, global developmental delay. *N*, number of observations; %, frequency

Table 2 presents the demographic characteristics of the caregivers and children. The majority of caregivers were females, mainly mothers of the child, and housewives. Most caregivers were married, living in urban areas, and of Orthodox Christian religion. Most did not receive any education above the primary school level. The mean age of caregivers at the time of the interview was of 34 years (s.d. = 7.1).

**Table 2.** Demographic information

	Control		Case		Rest*	
	139		139		22	
Total	*N*	%	*N*	%	*N*	%
***Caregiver***						
Gender***						
Male	43	30.9	14	10.1	7	31.8
Female	96	69.1	125	89.9	15	68.2
Marital Status						
Married	122	87.8	111	79.9	18	81.8
Single	4	2.9	5	3.6	1	4.6
Divorced	10	7.2	18	13.0	3	13.6
Widowed	3	2.2	4	2.9	0	0
Missing	0	0	1	0.7	0	0
Residence***						
Rural	45	32.4	18	13.0	6	27.3
Urban	91	65.5	118	84.9	16	72.7
Missing	3	2.2	3	2.2	0	0
Religion**						
Orthodox Christian	95	68.4	79	56.8	15	68.2
Protestant	18	13.0	11	7.9	1	4.6
Catholic	1	0.7	1	0.7	0	0
Muslim	23	16.6	47	33.8	6	27.3
Other *(Please Specify)*	2	1.4	0	0	0	0
Missing	0	0	1	0.7	0	0
Relationship to child***						
Mother	94	67.6	116	83.5	14	63.6
Father	41	29.5	13	9.4	6	27.3
Extended family	4	2.9	5	3.6	2	9.1
Other	0	0	3	2.2	0	0
Missing	0	0	2	1.4	0	0
Education						
No formal education	20	14.4	20	14.4	4	18.2
Primary school *(Grade 1-8)*	49	35.3	42	30.2	12	54.6
Secondary school *(Up To Grade 10* *+* *2 Or 12* *+* *1)*	46	33.1	47	33.8	4	18.2
Diploma *(Grade 10* *+* *3 And Above Or 12* *+* *2 And Above)*	12	8.6	11	7.9	1	4.6
College	9	6.5	17	12.2	0	0
Missing	3	2.2	2	1.4	1	4.6
Occupation*						
Farmer	7	5	5	3.6	4	18.2
Housewife	41	29.5	72	51.8	13	59.1
Merchant	15	10.8	16	11.5	2	9.1
Student	1	0.7	0	0	0	0
Civil servant	11	7.9	6	4.3	1	4.6
Daily labourer	19	13.7	11	7.9	0	0
Other	8	5.8	4	2.9	0	0
Missing	37	26.6	25	18	2	9.1
	Mean (s.d.)	Range	Mean (s.d.)	Range	Mean (s.d.)	Range
Age (years)	33.4 (6.7)	17–63	34.9 (7.6)	18–62	34.6 (6.8)	22–50
Number of children under care	2.4 (1.2)	1–8	2.4 (1.2)	1–10	3.2 (1.8)	1–8
***Child***						
Gender***						
Male	69	49.6	104	74.8	13	59.1
Female	69	49.6	35	25.2	9	40.9
Missing	1	0.7	0	0	0	0
	Mean (s.d.)	Range	Mean (s.d.)	Range	Mean (s.d.)	Range
Age (years)***	4.3 (2.0)	2–9	5.3 (1.8)	2–9	5.6 (2.0)	2–9

*Note:* ^$^Rest group refers to 22 participants included in the EFA analyses that did not meet inclusion criteria for either the case or control group. Significant differences between cases and controls are displayed as *p < 0.05, **p < 0.01, ***p < 0.001; *N*, number of observations; %, frequency; s.d., standard deviation.

Statistically significant differences between cases and controls were observed for the caregivers' gender (*p* < 0.001), with a higher proportion of females among cases, and residence (*p* < 0.001), with more cases living in urban areas. Furthermore, we report significant differences in religion, (*p* = 0.004), observing a higher proportion of Muslims among cases compared to controls, relationship to the child (*p* < 0.001), where caregivers in the case group were more often mothers, and occupation (*p* = 0.02), with a higher proportion of housewives among cases.

62% of children were male, with an average age of 4.9 years (s.d. = 2.0). Among children, comparison across cases and controls showed significant differences in age (*p* < 0.001), with cases being older than controls, and gender (*p* < 0.001), with a higher proportion of males in the case group in line with the observation that NDDs are more common in boys (Loomes *et al*., 2017; Sayal *et al*., 2018). The retest sample consisted of *N* = 40 caregivers (*N* = 19 cases, *N* = 20 controls, *N* = 1 rest).

Items *i ‘Sign language’, j ‘Speaking English’,* and *m ‘Other’* from the CP-A-mode, had a median and iqr of 0 across test and retest, suggesting no or limited variability. These items were therefore removed. The remaining *N* = 10 items for CP-A-mode and *N* = 23 items for CP-A-function were further analysed.

#### Exploratory factor analysis

The Scree-test and eigenvalues (online Supplementary material Fig. S2) suggested a 2-factor solution for the CP-A-mode (*χ*^2^ = 1501.84, *p* < 0.001). The correlations between items and promax-rotated common factors are displayed in Table 3. Factor loadings and structure matrix indicated that items *a* ‘*Facial expression’, c ‘Gestures’, d ‘Body movements’, e ‘Looking or use of eye gaze’, f ‘Pointing’,* and *h ‘Showing you objects’* loaded more strongly on factor 1. Items *b* ‘*Making noises (vocals)’, g ‘Showing you pictures’, j ‘Speaking Amharic’* and *l ‘Behaviour’,* instead loaded on factor 2. Factor 1 generally corresponds to *physical communication*, while factor 2 to *verbal communication*. Item *g ‘Showing you pictures’* presented moderate loadings for both factors (slightly higher for the *verbal communication*) that were maintained throughout different rotation methods. Item *l* had a negative loading on *verbal communication*, indicating that caregivers of children with poor verbal communication tended to highlight the use of behaviour as the main mode of their child's communication.

**Table 3.** Factor loadings of the items of the CP-A-mode and CP-A-function

Communicative Mode			
Item		Factor 1 *Physical communication*	Factor 2 *Verbal communication*
	*1. How does (child's name) communicate with you?*		
	a. Facial expression	0.55	0.53
	b. Making noises *(vocals)*	0.33	0.61
	c. Gestures	0.73	0.37
	d. Body movements	0.65	0.40
	e. Looking or use of eye gaze	0.67	0.58
	f. Pointing	0.79	0.27
	g. Showing you pictures	0.50	0.59
	h. Showing you objects	0.72	0.22
	j. Speaking *Amharic, Guragigna meskan and Mareko*	0.40	0.89
	l. Behaviour	−0.17	−0.71
Communicative function			
Item		Factor 1 *Communicative function*	
*Expressive*	1. Does (child's name) let you know if s/he doesn't like something you are giving him/her?	0.78	
	2. Does (child's name) let you know when s/he is sad or upset?	0.73	
	3. Does (child's name) indicate ‘yes’, for example, if s/he wants to do something?	0.88	
	4. Does (child's name) indicate ‘no’, for example, if s/he doesn't want to do something?	0.83	
	5. Does (child's name) let you know if s/he is happy?	0.81	
	6. Does (child's name) let you know if s/he is not happy?	0.77	
	7. Does (child's name) get your attention when s/he wants?	0.50	
	8. Does (child's name) tell you what s/he wants, for example, food or drink?	0.80	
	9. Does (child's name) greet people?	0.80	
	10. Does (child's name) get you to do something again which s/he has just enjoyed doing?	0.70	
	11. Does (child's name) ask for help when s/he can't manage to do something by him/herself?	0.61	
	12. Does (child's name) comment on things that are happening	0.87	
	13. Does (child's name) ask simple questions, for example, what is it or where's mummy?	0.88	
	14. Does (child's name) tell you about something that has happened, for example, when you weren't looking?	0.90	
*Social*	1. Does (child's name) start up communication with people in the family?	0.87	
	2. Does (child's name) start up communication with other people?	0.88	
	3. Does (child's name) try again if you don't understand him/her?	0.82	
	4. Does (child's name) communicate with other people in a way that is polite?	0.89	
*Receptive*	1. Does (child's name) understand when you tell him/her ‘no’?	0.79	
	2. Does (child's name) understand simple instructions?	0.87	
	3. Does (child's name) understand if you ask for something that is not in the immediate environment?	0.87	
	4. Does (child's name) understand if you communicate about something that is going to happen or has already happened?	0.86	
	5. Does (child's name) understand stories?	0.88	

*Note:* Factor loadings extracted through principal axis factoring. Shaded cells indicate factor allocation.

For communicative function, the Scree-test and eigenvalues (online Supplementary material Fig. S3) suggested a 1-factor solution. The model had a good fit to the data (*χ*^2^ = 8784.87; *p* < 0.001), suggesting that the function items assessed a single construct. All items loaded positively on the factor, see Table 3.

Similarly, for the CP-A-total the Scree-test and eigenvalues (online Supplementary material Fig. S4) indicated a 1-factor solution. The model had a good fit to the data (*χ*^2^ = 2.1e + 04; *p* < 0.001), suggesting that all items of the analysed CP-A scales can be meaningfully subsumed under a single construct reflecting overall communication activities. All items, besides Item *l,* loaded positively on the factor (online Supplementary material Table S4).

### Validity

#### Within-scale validity

A summary of the psychometric properties of the CP-A is provided in Table 4. The Spearman's rank correlation coefficient (*ρ*) between the CP-A-mode's *verbal communication* and *physical communication* scores was significant and moderately positive (*ρ* = 0.41, *p* < 0.001). Similarly, the correlation between the CP-A-mode and CP-A-function scores was significant and moderately positive (*ρ* = 0.59, *p* < 0.001), further justifying the adoption of the CP-A-total as a unidimensional summary scale (Lamping *et al*., 2002; Dancey and Reidy, 2017).

**Table 4.** Psychometric properties of the CP-A's identified factors

		CP-A-mode's physical communication	CP-A-mode's verbal communication	CP-A-function	CP-A -total
*Psychometric property*	*Constituent parts*		*N* *=* *300*		
*Validity*	Known-group validity (Δ mean)	2.89***^a^	2.09***^a^	28.94***^a^	33.93***^a^
	Convergent validity (*ρ*)	−0.24*	−0.27*	−0.23 NS	−0.25*
*Reliability*	Internal Consistency (Cronbach's alpha)	0.80^b^	0.72^c^	0.96^d^	0.96^e^
	Test-retest reliability (Weighted Kappa)	0.60–0.86***	0.65–0.76***	0.60–0.95***	0.60–0.95***
	Test-retest reliability (Interclass Correlation Coefficient)	0.81***	0.83***	0.96***	0.97***

*Note:* Significant differences between cases and controls are displayed as *p < 0.05, **p < 0.01, ***p < 0.001; *N*, number of participants.

a Unadjusted mean difference in summative scores (controls minus cases).

b 6 items.

c 4 items.

d 23 items.

e 33 items.

#### Known-group validity

The multiple regression analyses indicate that clinical controls scored significantly higher than cases on CP-A-mode's *physical communication* (Δmean = 2.89, *p* < 0.001) and *verbal communication* (Δmean = 2.09, *p* < 0.001). This holds true also for the CP-A-function (Δmean = 28.94, *p* < 0.001) and CP-A-total (Δmean 33.93, *p* < 0.001), Table 5. The significant group differences persisted when adjusting for covariates (caregiver's age, religion, occupation, residence, and relationship to the child; children's age and gender). Cohen's *d* estimates for CP-A-mode's factors were medium when unadjusted and small when accounting for covariates. Estimates were large for CP-A-function and CP-A-total across both scenarios (Cohen, 1988).

**Table 5.** Adjusted and unadjusted mean differences in summative scores between cases and controls

				Unadjusted				Adjusted			
	*N*	mean (s.d.) cases	mean (s.d.) controls	Δ mean	95% CI		Cohen's *d*	Δ mean	95% CI		Cohen's *d*
*CP-A-mode's physical communication*	6	11.21 (5.84)	14.10 (4.04)	2.89***	(1.71	4.08)	0.58	2.25**	(0.62	3.89)	0.33
*CP-A-mode's verbal communication*	4	6.85 (3.18)	8.94 (2.16)	2.09***	(1.45	2.73)	0.77	1.61***	(0.69	2.52)	0.42
*CP-A-function*	23	36.67 (19.81)	65.61 (17.31)	28.94***	(24.55	33.34)	1.56	30.42***	(24.60	36.23)	1.24
*CP-A-total*	33	54.73 (25.82)	88.65 (19.77)	33.93***	(28.49	39.36)	1.48	34.28***	(26.93	41.62)	1.10

*Note*: Significant differences between cases and controls are displayed as *p < 0.05, **p < 0.01, ***p < 0.001; Δ mean, mean difference (controls minus cases); *N*, number of items. Mean differences unadjusted or adjusted for covariates.

#### Convergent validity

There was a significant correlation between clinical severity and CP-A-mode's *physical communication* (*ρ* = −0.24, *p* = 0.04) as well as *verbal communication* (*ρ* = −0.27, *p* = 0.02). Similar results were obtained for the CP-A-total (*ρ* = −0.25, *p* = 0–04). For the CP-A-function, the point estimate of the correlation was in the same direction and of similar magnitude (*ρ* = −0.23) but was not significant (*p* = 0.59) (Dancey and Reidy, 2017).

### Reliability

The internal consistency for the CP-A-mode factors was acceptable (*physical communication:* 4 items, *α* = 0.80, 95% confidence interval (CI) ⩾ 0.78; *verbal communication:* 6 items, *α* = 0.72, 95% CI ⩾ 0.69). Excellent internal consistencies were obtained for the CP-A-function (23 items, *α* = 0.96, 95% CI ⩾ 0.96) and the CP-A-total (33 items, *α* = 0.97, 95% CI ⩾ 0.96). Assessing internal consistency separately for cases and controls suggested that levels remained acceptable for the CP-A-mode's *physical communication* (cases: *α* = 0.78, 95% CI ⩾ 0.74; controls: *α* = 0.78, 95% CI ⩾ 0.74), but were low for *verbal communication* (cases: *α* = 0.63, 95% CI ⩾ 0.57; controls: *α* = 0.64, 95% CI ⩾ 0.57). The internal consistency remained excellent for CP-A-function (cases: *α* = 0.94, 95% CI ⩾ 0.93; controls: *α* = 0.96, 95% CI ⩾ 0.95) and CP-A-total (cases: *α* = 0.94, 95% CI ⩾ 0.95; controls: *α* = 0.94, 95% CI ⩾ 0.93) (George and Mallery, 2010; Revicki, 2014).

Weighted Kappa coefficients ranged between 0.60 and 0.86 for *physical communication* and between 0.65 and 0.76 for *verbal communication* (*p* < 0.001), demonstrating moderate to a substantial agreement among items. For CP-A-function and CP-A-total, the agreement was moderate to near perfect for all items (min = 0.60, max = 0.95; p < 0.001) (McHugh, 2012; Portney, 2020). Please refer to online Supplementary material Tables S5, S6 for details.

The ICC indicated good test-retest reliability for the CP-A-mode's *physical communication* (ICC = 0.81, 95% CI 0.67–0.89; *p* < 0.001) and *verbal communication* (ICC = 0.83; 95% CI 0.70–0.91; *p* < 0.001). Excellent test-retest reliability was observed for the CP-A-function (ICC = 0.96; 95% CI 0.94–0.98; *p* < 0.001) and CP-A-total (ICC = 0.97; 95% CI 0.95–0.98; *p* < 0.001) (Koo and Li, 2016; Portney, 2020).

## Discussion

This paper reports the first validation study of the CP-A, a caregiver-reported tool for the assessment of children's communication. Our aim was to address the need for a psychometrically sound, brief, and culturally appropriate outcome measure for use in Ethiopia. We investigated two sections of the CP-A: communicative mode and function. Within communicative mode we identified two factors, *verbal* and *physical communication*; the communicative function scale was unidimensional. EFA results indicated that all items, from both scales, can be meaningfully summarised into one single factor. This suggested the adoption of a summary score (CP-A-total), supported by findings of strong correlations between the identified factors. As hypothesised, children with NDDs (cases) scored lower than clinical controls. Moreover, scale scores were negatively correlated to clinical severity ratings of NDDS, indicating that children with more severe NDDs used fewer modes of communication and applied fewer functions of communication. We observed acceptable to excellent internal consistency as well as test-retest reliability. Overall, these results demonstrated the validity and reliability of the CP-A-mode, function, and communication-total scales.

Within the CP-A-mode, for items *i ‘Sign language’, j ‘Speaking English’,* and *m ‘Other’,* most responses were equal to 0 (i.e. ‘never’), suggesting these items have little relevance in assessing communicative mode within our sample. For item *i* this is likely due to the fact that none of our participants was reported to have hearing loss. Furthermore, formal sign language has received very limited implementation in Ethiopia so far (Wakuma, 2015), and caregivers could have selected item c ‘*Gestures*’ to indicate informal signs as modes of communication. The lack of variability in item *j* can be attributed to the fact that English is not widely spoken across the population (Central Intelligence Agency, 2020). Most caregivers answered ‘never’ to item *m ‘Other’,* suggesting that all previously administered questions had exhaustively described the communicative modes adopted by their children. For these reasons, items *i, j*, and *m* of the communicative mode were dropped from subsequent analyses. The CP-A-function scale was retained in its entirety.

For communicative mode, we found support for a 2-factor structure. All factor 1 items fit with the profile for *physical communication*. For factor 2, the construct of *verbal communication* is defined by the items with the highest loadings, *b* ‘*Making noises (vocals)’* and *j ‘Speaking Amharic’*. Item *g ‘Showing you pictures*’ showed more moderate loading, with substantial cross-loading on factor 1. Item *l ‘Behaviour’* had a strong negative correlation with factor 2, suggesting that caregivers of children who did not express themselves verbally were more likely to report their child's behaviour as a form of communication. This finding is in line with theoretical notions that see verbal acts and behaviours as equivalent in function (Carr and Durand, 1985). When verbal communication is severely impaired, behavioural expression may become challenging to the person and others (Royal College of Psychiatrists and Banks, 2007). The occurrence of challenging behaviours (e.g. self-injury, stereotypy) is reported across NDDs and cultural contexts (McClintock *et al*., 2003; Adeniyi and Omigbodun, 2016; O'Nions *et al*., 2018). The Amharic version of the item, contrary to the English original, was followed by examples of challenging behaviours in brackets ‘(e.g. crying and shouting)’. These examples may have contributed to caregivers' interpretation of the item as primarily concerning challenging behaviours rather than behaviour overall.

Clinical controls scored higher than cases on the CP-A-mode, function, and communication-total scales, even after adjusting for covariates. This supports both our initial hypothesis and that of the developers of the measure: these scales were designed to reflect higher perceived competence in communication through higher summative scores (Bunning *et al*., 2014). This is in line with studies investigating other tools assessing communication in higher-income countries (HICs), with lower ratings consistently indicating more profound impairments (Geurts *et al*., 2004; Norbury *et al*., 2004).

### Significance

This investigation represents the first exploration of the validity and psychometric properties of the CP-A. Compared to other measures, it is more suitable for application in low-resource settings. Unlike caregiver-reported tools developed in Western HICs that assess similar constructs, the CP-A is free and open access, and this avoids the significant costs and adaptation negotiations associated with copyright-restricted instruments (Durkin *et al*., 2015). Moreover, instruments developed in Western HICs often require extensive adaptations to be suitable in non-Western lower-income contexts (Marlow *et al*., 2019; de Leeuw *et al*., 2020) As the CP-A is one of the very rare measures developed in an LMIC (Bunning *et al*., 2014; Goldfeld and Yousafzai, 2018), it does not encounter these issues. Its design and content are more likely to be relevant and appropriate for the African context. Nevertheless, adaptations of limited entities (e.g. referring to the languages spoken locally) are required to fit the specific context of an application. These must be paired with further explorations of the psychometric properties in diverse settings across the continent,

The only previously published research using the CP-A as an outcome measure investigated the impact of a caregiver-driven intervention for children with complex communication needs in rural Kenya (Bunning *et al*., 2014). While the sample size of this study was small (*N* = 10) and did not include a control group, results suggested sensitivity to change, as scores in the activities for communication sections, including communicative mode and function, were significantly higher post-intervention compared to pre-intervention (Bunning *et al*., 2014). The integration of their findings with that of our investigation demonstrates the potential of the CP-A for use in interventional studies. Adopting an accessible and appropriate measure like the CP-A across investigations on NDDs would increase the comparability of results, aiding the evaluation and implementation of effective interventions in low-resource settings.

### Limitations

Limitations should be considered when evaluating our results. Significant differences were reported for some demographic variables, especially in terms of age, where our clinical control group was younger than the case group. Nevertheless, it could be argued that the developmental age of the two groups is more comparable in this situation, given that the control group (with younger children that are naturally in the earlier phases of developing their communication abilities) still scores higher than the case group. The clinical severity assessment was carried out by general psychiatrists, with no specialist expertise in the assessment of child NDDs. Nevertheless, diagnosis and assessment of children with NDDs part of postgraduate training in psychiatry in Ethiopia and the psychiatrists involved in the study were experienced in making these diagnostic assessments. Psychiatrists used their clinical judgement rather than standardised tools to assess the severity of impairment of the children since there are no validated standardised clinical severity assessment scales available in Ethiopia to support the assessment of severity. Furthermore, the limited educational or supportive service provision available for children with NDDs in this setting means that reports from other professionals are not available to inform severity assessments. Thus severity is based on a single report from the caregiver during the clinical encounter and observations of the psychiatrist of the child in a clinical setting. Severity scores were collected for a small sample only, covering a limited range of complex communication needs. This study was conducted as part of a larger project focusing on NDDs. Most cases included in our study had ASD or ID, rather than a wider group of complex communication needs and developmental disabilities for which the CP-A was also developed (e.g. sensory impairments). Lastly, participants were help-seeking families recruited in Addis Ababa. Our sample had an overrepresentation of urban families and may not be fully representative of the Ethiopian population.

### Future research

Further research could test our factor structure for the CP-A-mode and CP-A-function through confirmatory factor analysis. Recruitment of cases should be extended to a wider range of complex communication needs. In such studies, the relevance of items reflecting characteristics of developmental disabilities with no representation in our sample (i.e. *i ‘Sign language’*), should be re-evaluated. Future research should further examine whether the CP-A-mode, CP-A-function, and CP-A-total are sensitive to change induced by interventions. Lastly, future studies may also wish to consider other sections of the CP-A not included in the current evaluation.

## Conclusion

This work is the first investigation to explore the validity of CP-A, an open-access measure developed in and for the African context. The communication mode and function and their combined scales met the validity and reliability criteria as a measure for the assessment of caregiver-perceived activities for communication. We tested this among children with NDDs and concomitant complex communication needs in Ethiopia. We recommend the further validation of this scale. The CP-A has potential for application in intervention studies on NDDs across sub-Saharan Africa as a brief, quantitative, and culturally appropriate outcome measure.

## Supplementary material

For supplementary material accompanying this paper visit http://dx.doi.org/10.1017/gmh.2021.44. click here to view supplementary material
