# Supplementary material for: Validation of the Communication Profile-Adapted in Ethiopian children with neurodevelopmental disorders
Source: Glob Ment Health (Camb). 2021 Dec 13;8:e45. doi: 10.1017/gmh.2021.44 (PMC8679817; doi:10.1017/gmh.2021.44)
Supplement: Supplementary file 1 [file S2054425121000443sup001.docx]

**Supplementary material**

**Table S1 –** Clinical severity scores for the assessed subgroup of children diagnosed with ID and/or ASD

|  | | ID | |  | | ASD | |  | Combined |  |
| --- | --- | --- | --- | --- | --- | --- | --- | --- | --- | --- |
|  | | N | | % | | N | | % | N | % |
| Total | | 24 | |  | | 50 | |  | 91 |  |
| Severity |  | |  | |  | |  | |  |  |
| Mild | | 11 | | 45.8 | | 19 | | 38 | 28 | 40 |
| Moderate | | 8 | | 33.3 | | 27 | | 54 | 34 | 48.6 |
| Severe | | 5 | | 20.8 | | 4 | | 8 | 8 | 11.4 |

*Note. N: number of observations; %: frequency.*

**Table S2 –** Main physical health problems reported in the clinical control group and examples of specific conditions

| Physical health conditions (N=139) |  |
| --- | --- |
| *Cardiovascular* | congenital health failure, rheumatic heart disease |
| *Respiratory* | asthma, bronchitis, community-acquired pneumonia, pertussis, unspecified respiratory tract infection |
| *Neurological* | traumatic brain injury, stroke, hemiparesis |
| *Other* | moderate-severe acute malnutrition, meningitis, hepatitis, tuberculosis |

*Note. We report a list of conditions rather than a frequency table given the high levels of comorbidities reported in the sample*

**Table S3 –** Conditions and missing data reported in the rest group.

|  | Rest (N=22) |  |  |
| --- | --- | --- | --- |
|  |  | N | % |
| *Condition* | Behavioural problems / disturbances | 1 | 4.5 |
|  | Behavioural problems / disturbances + epilepsy / seizure disorder | 2 | 9.1 |
|  | Brief psychosis + PTSD | 1 | 4.5 |
|  | Bipolar Disorder | 1 | 4.5 |
|  | Childhood onset schizophrenia | 2 | 9.1 |
|  | Major depressive disorder | 1 | 4.5 |
|  | Not otherwise specified mental disorder | 1 | 4.5 |
|  | Epilepsy / seizure disorder | 8 | 36.4 |
| *Missing diagnostic information* |  | 5 | 22.7 |
|  | **Total** | 22 | 100 |

*Note: Rest group refers to 22 participants included in the EFA analyses that did not meet inclusion criteria for either the case or control group*


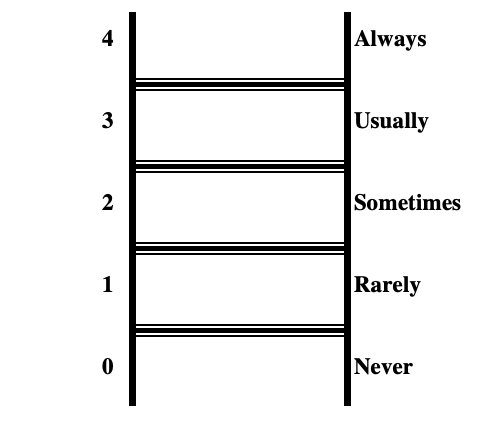


**Figure S1–**Adapted response ladder scale participants referred to when answering items from the CP-A

 **Figure S2 –** Scree plot for the CP-A-mode; the red line indicates an eigenvalue of 1

**Figure S3–** Scree plot for CP-A-function; the red line indicates an eigenvalue of 1

**Figure S4–** Scree plot for the CP-A-total; the red line indicates an eigenvalue of 1

**Table S4 -** Factor loadings for the CP-A-total

| Item | | Factor 1  *CP-A-total* | |
| --- | --- | --- | --- |
| CP-A-mode |  | |  |
|  | *1. How does (child’s name) communicate with you?* | |  |
|  | a. Facial expression | | 0.54 |
|  | b. Making noises *(vocals)* | | 0.49 |
|  | c. Gestures | | 0.44 |
|  | d. Body movements | | 0.43 |
|  | e. Looking or use of eye gaze | | 0.58 |
|  | f. Pointing | | 0.39 |
|  | g. Showing you pictures | | 0.57 |
|  | h. Showing you objects | | 0.29 |
|  | j. Speaking *Amharic, Guragigna meskan and Mareko* | | 0.89 |
|  | l. Behaviour | | -0.66 |
| CP-A-function |  | |  |
| *Expressive* | 1. Does (child’s name) let you know if s/he doesn’t like something you are giving him/her? | | 0.78 |
|  | 2. Does (child’s name) let you know when s/he is sad or upset? | | 0.71 |
|  | 3. Does (child’s name) indicate ‘yes’, for example, if s/he wants to do something? | | 0.88 |
|  | 4. Does (child’s name) indicate ‘no’, for example, if s/he doesn’t want to do something? | | 0.84 |
|  | 5. Does (child’s name) let you know if s/he is happy? | | 0.80 |
|  | 6. Does (child’s name) let you know if s/he is not happy? | | 0.76 |
|  | 7. Does (child’s name) get your attention when s/he wants? | | 0.51 |
|  | 8. Does (child’s name) tell you what s/he wants, for example food or drink? | | 0.80 |
|  | 9. Does (child’s name) greet people? | | 0.82 |
|  | 10. Does (child’s name) get you to do something again which s/he has just enjoyed doing? | | 0.70 |
|  | 11. Does (child’s name) ask for help when s/he can’t manage do something by him/herself? | | 0.61 |
|  | 12. Does (child's name) comment on things that are happening | | 0.85 |
|  | 13. Does (child’s name) ask simple questions, for example, what is it or where’s mummy? | | 0.87 |
|  | 14. Does (child's name) tell you about something that has happened, for example, when you weren’t looking? | | 0.88 |
| *Social* | 1. Does (child’s name) start up communication with people in the family? | | 0.88 |
|  | 2. Does (child’s name) start up communication with other people? | | 0.88 |
|  | 3. Does (child’s name) try again if you don’t understand him/her? | | 0.82 |
|  | 4. Does (child’s name) communicate with other people in a way that is polite? | | 0.89 |
| *Receptive* | 1. Does (child’s name) understand when you tell him/her ‘no’? | | 0.80 |
|  | 2. Does (child’s name) understand simple instructions? | | 0.88 |
|  | 3. Does (child's name) understand if you ask for something that is not in the immediate environment? | | 0.86 |
|  | 4. Does (child's name) understand if you communicate about something that is going to happen or has already happened? | | 0.85 |
|  | 5. Does (child's name) understand stories? | | 0.88 |

*Note: Factor loadings extracted through principal axis factoring.*

**Table S5 -** Weighted Kappa results for communicative mode.

| Item | Agreemsent | Expected agreement | Kappa | 95% CI | Std. Err. | Z | Prob>Z |
| --- | --- | --- | --- | --- | --- | --- | --- |
| a. Facial expression | 90% | 72.7% | 0.63 | 0.48-0.79 | 0.10 | 6.42 | 0.000 |
| b. Making noises *(vocals)* | 89.4% | 57.9% | 0.75 | 0.65-0.85 | 0.12 | 6.28 | 0.000 |
| c. Gestures | 87.5% | 68.8% | 0.60 | 0.43-0.76 | 0.10 | 5.97 | 0.000 |
| d. Body movements | 90% | 68.2% | 0.69 | 0.53-0.84 | 0.11 | 6.38 | 0.000 |
| e. Looking or use of eye gaze | 95% | 65% | 0.86 | 0.78-0.94 | 0.12 | 7.19 | 0.000 |
| f. Pointing | 89.4% | 71.4% | 0.63 | 0.47-0.78 | 0.11 | 5.98 | 0.000 |
| g. Showing you pictures | 87.5% | 64.6% | 0.65 | 0.50-0.80 | 0.12 | 5.50 | 0.000 |
| h. Showing you objects | 90.6% | 68.3% | 0.70 | 0.56-0.85 | 0.10 | 6.86 | 0.000 |
| j. Speaking *Amharic, Guragigna meskan and Mareko* | 90% | 57.8% | 0.76 | 0.62-0.90 | 0.13 | 5.90 | 0.000 |
| l. Behaviour | 91.3% | 68.2% | 0.73 | 0.59-0.86 | 0.12 | 6.20 | 0.000 |

**Table S6 -** Weighted Kappa results for communicative function.

| Item | | Agreement | Expected agreement | Kappa | 95% CI | Std. Err. | Z | Prob>Z |
| --- | --- | --- | --- | --- | --- | --- | --- | --- |
| *Expressive* | 1. Does (child’s name) let you know if s/he doesn’t like something you are giving him/her? | 89.2% | 73.3% | 0.60 | 0.44-0.71 | 0.12 | 5.05 | 0.000 |
|  | 2. Does (child’s name) let you know when s/he is sad or upset? | 89.2% | 71.3% | 0.62 | 0.45-0.75 | 0.12 | 5.32 | 0.000 |
|  | 3. Does (child’s name) indicate ‘yes’, for example, if s/he wants to do something? | 93.1% | 55.5% | 0.85 | 0.76-0.93 | 0.13 | 6.76 | 0.000 |
|  | 4. Does (child’s name) indicate ‘no’, for example, if s/he doesn’t want to do something? | 95.8% | 54.7% | 0.91 | 0.87-0.97 | 0.13 | 7.23 | 0.000 |
|  | 5. Does (child’s name) let you know if s/he is happy? | 86.3% | 59.1% | 0.66 | 0.52-0.81 | 0.13 | 5.31 | 0.000 |
|  | 6. Does (child’s name) let you know if s/he is not happy? | 90.6% | 72.8% | 0.66 | 0.52-0.79 | 0.11 | 5.93 | 0.000 |
|  | 7. Does (child’s name) get your attention when s/he wants? | 90.6% | 75.4% | 0.62 | 0.46-0.78 | 0.10 | 6.15 | 0.000 |
|  | 8. Does (child’s name) tell you what s/he wants, for example food or drink? | 86.7% | 59% | 0.67 | 0.54-0.81 | 0.11 | 6.04 | 0.000 |
|  | 9. Does (child’s name) greet people? | 91.3% | 63.9% | 0.76 | 0.65-0.87 | 0.11 | 7.18 | 0.000 |
|  | 10. Does (child’s name) get you to do something again which s/he has just enjoyed doing? | 93.1% | 73% | 0.75 | 0.62-0.87 | 0.11 | 6.72 | 0.000 |
|  | 11. Does (child’s name) ask for help when s/he can’t manage do something by him/herself? | 87.5% | 67.8% | 0.61 | 0.47-0.76 | 0.10 | 5.85 | 0.000 |
|  | 12. Does (child's name) comment on things that are happening | 92.5% | 66.5% | 0.78 | 0.67-0.89 | 0.12 | 6.32 | 0.000 |
|  | 13. Does (child’s name) ask simple questions, for example, what is it or where’s mummy? | 93.8% | 58% | 0.85 | 0.77-0.94 | 0.13 | 6.80 | 0.000 |
|  | 14. Does (child's name) tell you about something that has happened, for example, when you weren’t looking? | 93.1% | 66.7% | 0.79 | 0.68-0.91 | 0.13 | 6.34 | 0.000 |
| *Social* | 1. Does (child’s name) start up communication with people in the family? | 91.9% | 71.8% | 0.71 | 0.61-0.82 | 0.12 | 5.97 | 0.000 |
|  | 2. Does (child’s name) start up communication with other people? | 89.4% | 64.3% | 0.70 | 0.58-0.82 | 0.11 | 6.29 | 0.000 |
|  | 3. Does (child’s name) try again if you don’t understand him/her? | 88.1% | 64.3% | 0.67 | 0.55-0.78 | 0.10 | 6.69 | 0.000 |
|  | 4. Does (child’s name) communicate with other people in a way that is polite? | 88.8% | 63.1% | 0.70 | 0.56-0.83 | 0.11 | 6.36 | 0.000 |
| *Receptive* | 1. Does (child’s name) understand when you tell him/her ‘no’? | 89.4% | 67.6% | 0.67 | 0.53-0.82 | 0.11 | 6.33 | 0.000 |
|  | 2. Does (child’s name) understand simple instructions? | 93.8% | 65.9% | 0.82 | 0.72-0.91 | 0.11 | 7.73 | 0.000 |
|  | 3. Does (child's name) understand if you ask for something that is not in the immediate environment? | 96.3% | 60.9% | 0.90 | 0.84-0.97 | 0.12 | 7.66 | 0.000 |
|  | 4. Does (child's name) understand if you communicate about something that is going to happen or has already happened? | 98.1% | 63.7% | 0.95 | 0.91-0.99 | 0.12 | 7.76 | 0.000 |
|  | 5. Does (child's name) understand stories? | 92.5% | 63% | 0.80 | 0.70-0.90 | 0.12 | 6.60 | 0.000 |
